# Fat oxidation rates and cardiorespiratory responses during exercise in different subject populations with post-acute sequelae of SARS-CoV-2 infection: a comparison with normative percentile values

**DOI:** 10.3389/fphys.2023.1310319

**Published:** 2023-12-08

**Authors:** Andrea Meloni, Roberto Codella, Daniel Gotti, Simone Di Gennaro, Livio Luzi, Luca Filipas

**Affiliations:** ^1^ Department of Biomedical Sciences for Health, Università degli Studi di Milano, Milan, Italy; ^2^ Department of Endocrinology, Nutrition and Metabolic Diseases, IRCCS MultiMedica, Milan, Italy; ^3^ Department of Neurosciences, Rehabilitation, Ophthalmology, Genetics and Mother-Child Sciences, Università degli Studi di Genova, Genova, Italy

**Keywords:** post-acute sequelae of SARS-CoV-2 infection, fat oxidation, metabolic dysfunction, cycling, exercise performance

## Abstract

**Introduction:** Post-acute sequelae of SARS-CoV-2 infection (PASC) presents a spectrum of symptoms following acute COVID-19, with exercise intolerance being a prevalent manifestation likely linked to disrupted oxygen metabolism and mitochondrial function. This study aims to assess maximal fat oxidation (MFO) and exercise intensity at MFO (FATmax) in distinct PASC subject groups and compare these findings with normative data.

**Methods:** Eight male subjects with PASC were involved in this study. The participants were divided into two groups: “endurance-trained” subjects ( $\dot{V}O_{2}\max$ > 55 mL/min/kg) and “recreationally active” subjects ( $\dot{V}O_{2}\max$ < 55 mL/min/kg). Each subject performed a graded exercise test until maximal oxygen consumption ( $\dot{V}O_{2}\max$ ) to measure fat oxidation. Subsequently, MFO was assessed, and FATmax was calculated as the ratio between $\dot{V}O_{2}$ at MFO and $\dot{V}O_{2}$ max.

**Results:** The MFO and FATmax of “endurance-trained” subjects were 0.85, 0.89, 0.71, and 0.42 and 68%, 69%, 64%, and 53%, respectively. Three out of four subjects showed both MFO and FATmax values placed over the 80th percentile of normative data. The MFO and FATmax of “recreationally active” subjects were 0.34, 0.27, 0.35, and 0.38 and 47%, 39%, 43%, and 41%, respectively. All MFO and FATmax values of those subjects placed below the 20th percentile or between the 20th and 40th percentile.

**Discussion:** Significant differences in MFO and FATmax values between ‘endurance-trained’ and “recreationally active” subjects suggest that specific endurance training, rather than simply an active lifestyle, may provide protective effects against alterations in mitochondrial function during exercise in subjects with PASC.

## Introduction

The syndrome of post-acute sequelae of SARS-CoV-2 infection (PASC) is a clinical condition characterized by a wide range of symptoms for 4 weeks or more following acute COVID-19 (World Health Organization, 2023). Exercise intolerance is one of the most widespread manifestations among subjects with PASC. This condition may be the result of impaired oxygen metabolic homeostasis and altered mitochondrial function (Astin et al., 2023). In general, mitochondrial disorders are a complex group of diseases caused by impairment of the mitochondrial respiratory chain (or electron transport chain), which in some patients can lead to an unexplained post-viral illness and myalgic encephalomyelitis/chronic fatigue syndrome (Wood et al., 2021). From a biochemical perspective, mitochondria may utilize various substrates depending on the load and duration of exercise (McArdle WD et al., 2001). During exercise lasting more than 1 minute, adenosine triphosphate (ATP) production is mainly generated via oxidative phosphorylation in the tricarboxylic acid cycle (TCA). The substrates utilized in the TCA to generate reducing equivalents fuel the electron transport chain inside mitochondria are fatty acids (FAs) and carbohydrates (CAs). In this energy production system, called the aerobic system, oxygen (O_2_) represents the final electron acceptor in mitochondria (McArdle WD et al., 2001). During a submaximal exercise, FA and CA are the main substrates to produce energy, and their contribution depends on exercise intensity. In this sense, rates of b-oxidation of FA (FATox rate) and CA oxidation rates (CHOox rate) are inversely proportional throughout an incremental exercise. Specifically, after reaching the maximal rate of fat oxidation (MFO), throughout an incremental test, the FATox rate tends to decrease, while the CHOox rate tends to increase. Thus, a more detailed analysis in the plasma of metabolites involved in the aerobic system was conducted recently in order to detect the presence of a metabolic dysfunction (Guntur et al., 2022). Results showed that the plasma of PASC subjects exhibited significantly higher levels of acyl-carnitines and free FAs and lower levels of pyruvate, lactate, and TCA metabolites such as succinate, malate, and citrate, compared to an healthy group and a group of subjects recovered from COVID-19, without PASC. These data are indicative of ongoing mobilization of FA but show impaired ability for oxidation due to mitochondrial dysfunction. In this contest, other research groups (de Boer et al., 2022) investigated whether patients with PASC had compromised mitochondrial function during graded exercise. A cardiopulmonary exercise test (CPET) has been used to calculate the FATox rate and lactate clearance, providing insight into mitochondrial function. Results showed inappropriately high arterial lactate levels and reduced FATox rate at relatively low exercise intensity in this type of subjects. Those data indicate that the transition from the FATox rate to the CHOox rate occurs earlier, suggesting, also in this case, dysfunctional mitochondria. Specifically, the premature lactate accumulation suggests either a metabolic shift in increased glycolysis or the inability to utilize lactate in the mitochondria as an alternative source of energy during exercise (de Boer et al., 2022). Normally, during an incremental exercise, lactate values and oxygen consumption ( $\dot{V}O_{2}$ ) increase, and the substrate contribution (FA and CA) can be estimated through the assessment of $\dot{V}O_{2}$ and carbon dioxide production ( $\dot{V}O_{2}$ ) (McArdle WD et al., 2001). Specifically, the FATox rate can be calculated by the stoichiometric equation (Frayn, 1983). Indeed, a well-trained endurance athlete is well known to have a high MFO and a declining FATox rate at exercise intensities of approximately 80%–85% of maximal oxygen consumption ( $\dot{V}O_{2}$ max) (Hurley et al., 1986; Martin et al., 1993; Phillips et al., 1996; Bircher and Knetchle, 2004). Moreover, fat oxidation capacity has been correlated with performance in ironman triathletes, which take part in ultra-endurance events (Frandsen et al., 2017). A recent review showed normative percentile values for MFO and FATmax in different subject populations in order to contextualize individually measured values and define the fat oxidation capacity of given research cohorts (Maunder et al., 2018). Given these findings, the objective of this study is to assess MFO and FATmax in two distinct populations of individuals with PASC and to compare these data with the normative percentile values from corresponding healthy cohorts. This analysis aims to determine whether the chronic effects of virus infection may impair FA metabolism during exercise in different subgroups of the PASC population.

## Materials and methods

Eight male subjects were involved in this study. The investigation lasted 3 months, and it was a pilot study that will serve as a basis for future more in-depth research on the topic at hand. Following the classification of a recent review (Maunder et al., 2018), regarding normative values of MFO and FATmax, subjects were divided into two groups: “endurance-trained” and “recreationally active.” “Endurance-trained” was defined as a subject with VO_2_max >55 mL/min/kg and active engagement in training for endurance events. “Recreationally active” was defined as physically active, not training for specific endurance events, and with VO_2_max <55 mL/min/kg (Maunder et al., 2018). Specifically, recreationally active subjects were involved in activities such as tennis, soccer, and gym fitness. Their anthropometric, physiological, and training characteristics are reported in Table 1. Before viral infection, both groups had been maintaining the same training or physical activity status compared to the post-COVID-19 period. The subjects had been reported a positive diagnosis of SARS-CoV-2 within 12 months before, with mild symptoms and no need for hospital care. The therapy was limited to taking NSAIDs for a few days. The subjects suffered PASC with preserved pulmonary and cardiac function and presented brain fog, insomnia, and memory impairment with the main symptoms. In order to detect the MFO and FATmax, each subject performed the “FATmax test” (Achten and Jeukendrup, 2004), a graded exercise test on an indoor roller (Direto XR-T, ELITE), with their own bike. Workload roller, expressed in watt (W), was monitored by software My E-Training, ELITE. Pedaling cadence was freely chosen and maintained constant ( ± five repetitions per minute (rpm)). Each participant completed the test at the same time of the day (±2 h) after 7–8 h of sleep and under similar environmental conditions (18–20 °C). Participants were asked to consume the same meals and drinks during the 24 h prior to testing and to fast overnight before the test. Specifically, after a free warm-up period of 5 min, the subjects performed a continuous ramp test for $\dot{V}O_{2}$ max, starting at 50 W and increasing 35 W every 2 min. At the end of each step, lactate was collected through a capillary blood sample. Each participant gave written informed consent prior to the study. Study procedures were conducted in accordance with the Declaration of Helsinki for experimentation on human participants. The study was approved by the Ethics Committee of the University of Milan (approval no. 52/20, attachment 4).

**TABLE 1:** Anthropometric, physiological, and training characteristics for each group. Data are reported as mean values ±SD.

Group	Age	Height (cm)	Weight (kg)	BMI (kg/m^2^)	$\dot{V}O_{2}$ max (mL/min/kg)	Endurance training (hours/week)	General physical activity (hours/week)
Endurance-trained	36.3 ± 15.3	175.8 ± 5.1	65.3 ± 7.6	21.1 ± 2.0	69.8 ± 14.9	14.3 ± 5.9	—
Recreationally active	26.5 ± 8.5	179.0 ± 8.2	74.1 ± 3.5	23.7 ± 1.8	47.5 ± 4.7	—	3 ± 1.4

### Gas exchange and lactate measurements

Expiratory ventilation (VE), $\dot{V}O_{2}$ , and $\dot{V}{\text{CO}}_{2}$ were analyzed using a portable metabolimeter (K5, COSMED). The highest 15-s VO2 was considered for the individual $\dot{V}O_{2}$ max assessment (Midgley et al., 2006; 2007). Lactate levels were determined by using a portable lactacidometer (Lactate Pro 2) through a capillary blood sample collected from the earlobe. Heart rate was monitored during the test with a heart rate sensor associated to the roller software (Polar H10; Polar Electro, Kempele, Finland). $\dot{V}O_{2}$ values were converted to associate power outputs by the linear regression of the $\dot{V}O_{2}$ vs. power output relationship using the last minute $\dot{V}O_{2}$ data in each incremental stage. For calculations of the total FATox rate during exercise, the stoichiometric equations by Frayn (1983) were used:

$$\text{FATox }\left[\frac{g}{\min}\right]=1.673\;x\;\dot{V}O_{2}\;\left[\frac{L}{\min}\right]-\;1.673\;x\;\dot{V}{\text{CO}}_{2}\;\left[\frac{L}{\min}\right].$$

FATox rates and $\dot{V}O_{2}$ for each step were assessed using the last minute $\dot{V}O_{2}$ data in each incremental step. Thus, MFO and FATmax, seen as the ratio between $\dot{V}O_{2}$ at MFO and $\dot{V}O_{2}$ max, were calculated. Furthermore, lactate kinetics throughout the test, until the workload step with the lowest FATox rate value, was determined for each subject. Thus, lactate values at MFO for each subject were reported.

## Results

**FIGURE 1:**
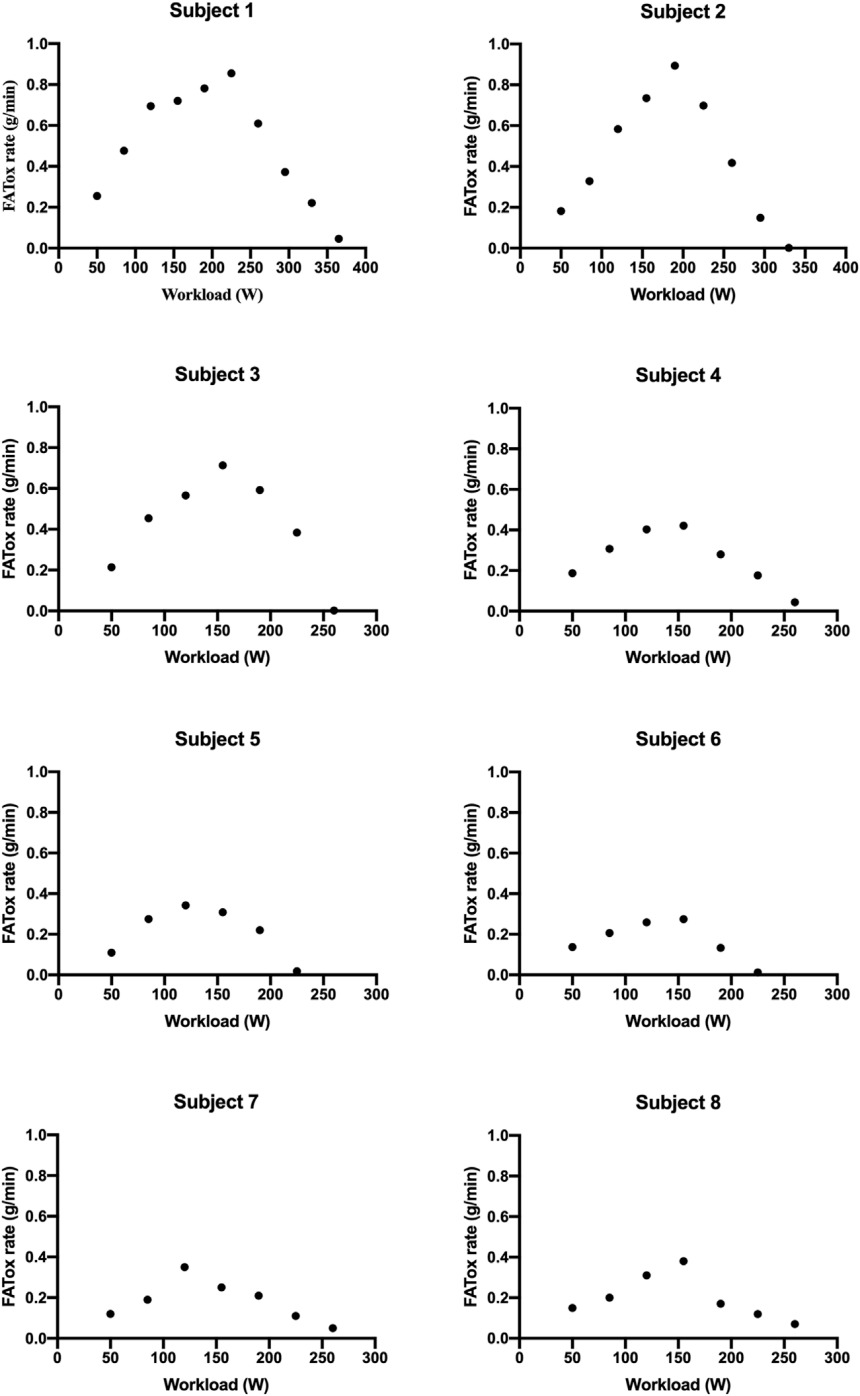
Subjects’ FATox rates for each step of the “FATmax test.”

**FIGURE 2:**
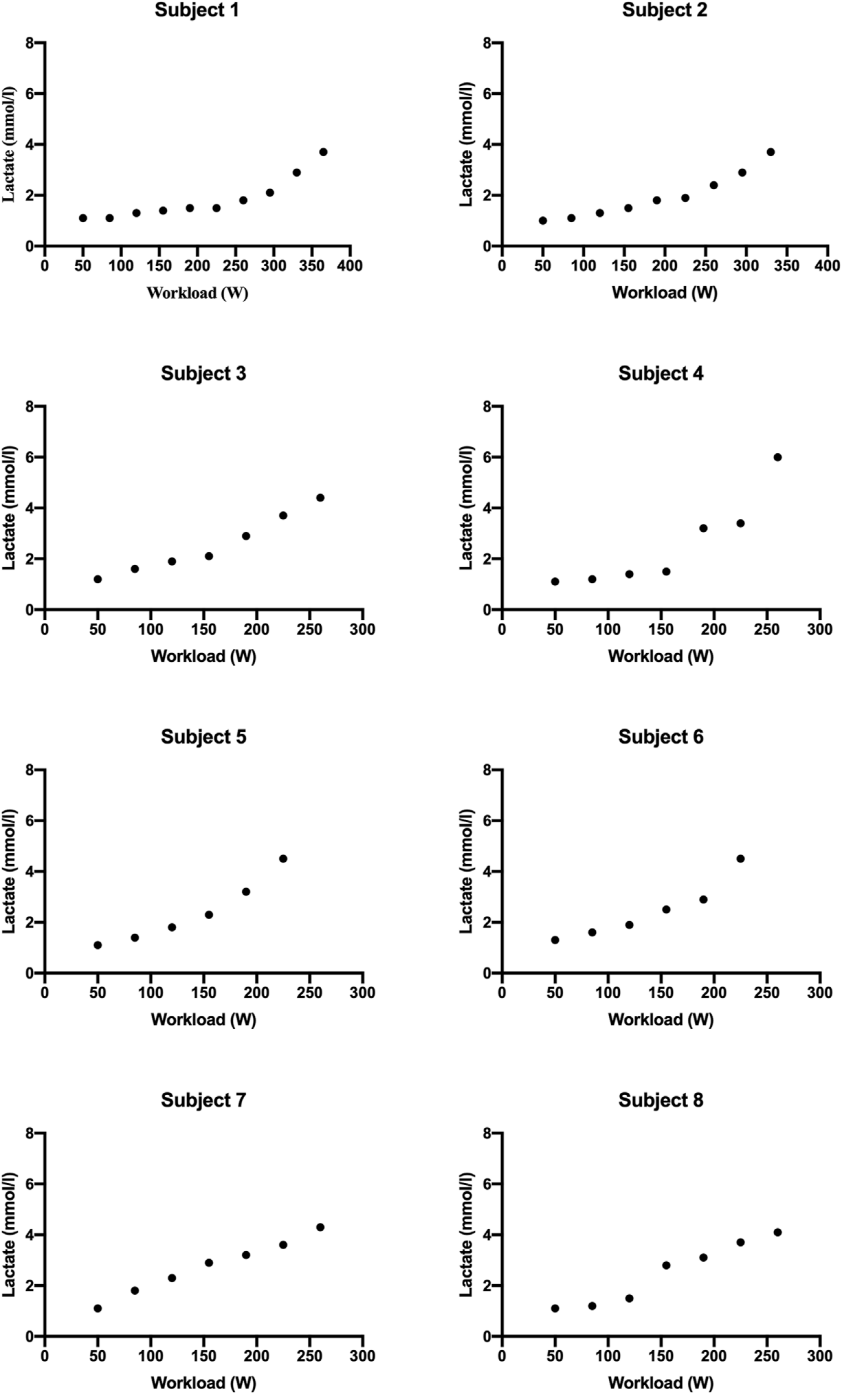
Lactate values for each subject until workload step with the lowest FATox rate.

MFO and FATmax values for “endurance-trained” and “recreationally active” subjects are summarized in Table 2. The subjects’ FATox rate for each step is described in Figure 1. Lactate kinetics until the workload step with the lowest FATox rate is described in Figure 2. The MFO and FATmax of “endurance-trained” subjects were 0.85, 0.89, 0.71, and 0.42 and 68%, 69%, 64%, and 53%, respectively. Subjects 1, 2, and 3 showed high MFO and FATmax, and those values both placed over the highest percentile (80th percentile) of normative data. MFO and FATmax of subject 4 placed between the 20th and 40th percentile of MFO normative data and between the 40th and 60th percentile of FATmax normative data. The MFO and FATmax of “recreationally active” subjects were 0.34, 0.27, 0.35, and 0.38 and 47%, 39%, 43%, and 41%, respectively. MFO and FATmax of subject 5 placed on the 20th percentile and between the 20th and 40th percentile, respectively. MFO and FATmax of subject 6 both placed below the 20th percentile. MFO and FATmax of subject 7 placed between the 20th and 40th percentile and below the 20th percentile, respectively. MFO and FATmax of subject 8 placed between the 20th and 40th percentile and below the 20th percentile, respectively. In the four “endurance-trained” subjects, lactate values at MFO were 1.5, 1.8, 2.1, and 1.5, respectively. In the four “recreationally active” subjects, lactate values at MFO were 1.8, 2.5, 2.3, and 2.8, respectively.

**TABLE 2:** MFO and FATmax values and relative percentile for each subject.

	Subject	$\dot{V}O_{2}$ max (mL/min/kg)	MFO	Relative percentile	FATmax (% $\dot{V}O_{2}$ max)	Relative percentile
**Endurance-trained**	1	81.2	0.85	>80th	68%	>80th
	2	84.1	0.89	>80th	69%	>80th
	3	56.7	0.71	>80th	64%	>80th
	4	57.0	0.42	20th–40th	53%	40th–60th
**Recreationally active**	5	41.0	0.34	20th	47%	20th–40th
	6	47.9	0.27	<20th	39%	<20th
	7	52.2	0.35	20th–40th	43%	<20th
	8	48.8	0.38	20th–40th	41%	<20th

## Discussion

PASC presents a spectrum of symptoms following acute COVID-19, with exercise intolerance being a prevalent manifestation likely linked to disrupted oxygen metabolism and mitochondrial function (Astin et al., 2023). Specifically, it seems that metabolic disfunction leads to a reduction of the FATox rate at relatively low exercise (de Boer et al., 2022). At the same time, it is well known that endurance training stimulates massive FA mobilization, and in this way, it is capable to improve both MFO and FATmax. Thus, the aim of this study is to analyze MFO and FATmax in different types of subjects with PASC and compare them with normative percentile values. All subjects presented mild symptoms of PASC with preserved pulmonary and cardiac function; thus, they were able to conduct a regular daily work activity. A recent review described normative data for different subject populations, including “endurance-trained,” engaged in training for endurance events, and “recreationally active,” physically active but not trained for endurance events (Maunder et al., 2018). In this context, this study showed how MFO and FATmax of subjects 3 from 4 of “endurance-trained” participants placed over the 80th percentile of their corresponding cohort. Only one “endurance-trained” subject presented MFO and FATmax values below the 60th percentile. These data could be the consequence of the infection of the subject by the virus two times within 6 months. Meanwhile, all “recreationally active” subjects showed MFO and FATmax values placed below the 40th percentile of their corresponding population. From lactate kinetics analysis, it appears how “endurance-trained” subjects show, generally, constant low values throughout the first steps of the test. Meanwhile, in the “recreationally active” group, there emerges, since the early steps, a more linear correlation between lactate values and workload. Indeed, lactate values at MFO in “endurance-trained” subjects were tendentially lower than in “recreationally active” subjects. These data strengthen the hypothesis that specific endurance training improves FA oxidation at relatively low exercise intensity, delaying, in this way, the increase of CHOox and, consequently, the accumulation of blood lactate. Taking into account all these preliminary findings, it could be suggestive of assuming that a training focus on endurance capacity may offer greater protection, compared to other types of exercise, against alterations caused by viral infection, such as mitochondrial dysfunction or, more broadly, anomalies within the pathway of FA oxidation.

## Limitations of this study and perspectives

Due to the absence of the subjects’ physiological data prior to SARS-CoV-2 infection, it was not possible comparing metabolic variables before and after infection in this preliminary report. In the future, several studies will be necessary to contribute to a deeper understanding of the relationship between exercise, mitochondrial function, and metabolic dysregulation in PASC subjects. Specifically, it might be useful to design a longitudinal study to investigate the effects of a structured exercise intervention, focusing on endurance training, on individuals with PASC and assess changes in exercise tolerance, mitochondrial function, and metabolic parameters over time. This study could include subgroups with varying exercise intensities and durations to determine the optimal training regimen for improving post-viral exercise intolerance in PASC patients. Additionally, it would be necessary conducting a comprehensive study to analyze metabolic profiles and mitochondrial function in different subtypes of PASC. Through this investigation, it will be possible to understand whether there are distinct metabolic signatures associated with varying symptomatology within PASC. This could involve advanced metabolomics profiling, including plasma metabolites and mitochondrial biomarkers, to identify specific metabolic dysfunctions in subgroups of PASC patients and tailor interventions accordingly. Finally, it could be interesting to compare the impact of different exercise modalities, such as aerobic exercise, resistance training, and a combination of both, on the metabolic and mitochondrial function as well. In this way, considerations for individualized exercise prescriptions based on the severity and nature of PASC symptoms would be valuable in developing targeted rehabilitation strategies.

## Data Availability

The raw data supporting the conclusion of this article will be made available by the authors, without undue reservation.
