# Corrigendum: Fat oxidation rates and cardiorespiratory responses during exercise in different subject populations with post-acute sequelae of SARS-CoV-2 infection: a comparison with normative percentile values

**DOI:** 10.3389/fphys.2024.1374886

**Published:** 2024-03-15

**Authors:** Andrea Meloni, Roberto Codella, Daniel Gotti, Simone Di Gennaro, Livio Luzi, Luca Filipas

**Affiliations:** ^1^ Department of Biomedical Sciences for Health, Università degli Studi di Milano, Milan, Italy; ^2^ Department of Endocrinology, Nutrition and Metabolic Diseases, IRCCS MultiMedica, Milan, Italy; ^3^ Department of Neurosciences, Rehabilitation, Ophthalmology, Genetics and Mother-Child Sciences, Università degli Studi di Genova, Genova, Italy

**Keywords:** post-acute sequelae of SARS-CoV-2 infection, fat oxidation, metabolic dysfunction, cycling, exercise performance

In the published article, there was an error in the **Funding** statement. It originally stated that the authors declared no financial support was received for the research, authorship, and/or publication of this article. The correct **Funding** statement appears below.

